# Comparison of Siglec-1 protein networks and expression patterns in sperm and male reproductive tracts of mice, rats, and humans

**DOI:** 10.14202/vetworld.2024.645-657

**Published:** 2024-03-21

**Authors:** Hazem Almhanna, Arun HS Kumar, David Kilroy, Gina Duggan, Jane A. Irwin, Bridget Hogg, Colm Reid

**Affiliations:** 1 Department of Anatomy and Histology, College of Veterinary Medicine, University of Al-Qadisiyah, Iraq; 2 School of Veterinary Medicine, University College Dublin, Belfield, Dublin-04, Ireland

**Keywords:** female reproductive tract, male reproductive tract, protein–protein interaction, sialic acid, sialic acid-binding immunoglobulin-like lectin-1

## Abstract

**Background:**

Sialic acid-binding immunoglobulin-like lectin 1 (Siglec-1) is a transmembrane glycoprotein involved in the sialic acid (Sia)-dependent regulation of the immune system. Siglec-1 expression has recently been identified in the male reproductive tract (MRT) of several species, including humans, cattle, horses, and sheep, and may play a role in modulating fertility in a Sia-dependent manner.

**Materials and Methods:**

In this study, protein–protein interaction (PPI) analysis of Siglec-1 was conducted to identify associated network protein conservation, and the expression of Siglec-1 in the MRT of mice and rats, including their accessory sex glands and spermatozoa was determined by immunostaining.

**Results:**

Network analysis of proteins with Siglec-1 in mice and rats demonstrated significant similarity to human Siglec-1 networks, suggesting a similar conservation of network proteins between these species and, hence, a potential conservation role in immune modulation and function. Specific immunostaining patterns of mouse and rat testes, epididymis, ductus deferens, accessory sex gland tissues, and sperm were detected using human Siglec-1. These results confirmed that the human Siglec-1 antibody could cross-react with mouse and rat Siglec-1, suggesting that the specific expression patterns of Siglec-1 in the MRT and sperm of both mice and rats are similar to those observed in other species.

**Conclusions:**

The conservation of Siglec-1 expression patterns in sperm and within the MRT and the similarity of protein networks for Siglec-1 across species suggest that Siglec-1 may function in a similar manner across species. These results also suggest that rodents may serve as a valuable model system for exploring the function of Siglecs in the reproductive system across species and their potential role in modulating fertility in a Sia-dependent manner.

## Introduction

Sialic acids (Sias) are nine-carbon sugars that are ubiquitous within the glycocalyx on the cell surface and abundant on the sperm plasma membrane [1–3]. Two parent forms, N-acetyl and N-glycolylneuraminic acid, can undergo extensive substitution, and over 50 different Sia forms have been described. Sia plays a role in cell protection and constitutes a complex array of ligands that are recognized by Sia-binding proteins [4] that are important for cell-to-cell communication and signaling.

Sialic acid-binding immunoglobulin-like lectins (Siglecs) are the largest group of Sia-binding proteins and are widely expressed and regulate adaptive and innate immune responses in macrophages and dendritic cells [5, 6]. Siglecs have a characteristic structural domain (V-set immunoglobulin-like domain) that recognizes specific Sia conformations. They also possess an extended region composed of a C2 immunoglobulin domain, a transmembrane region, and a cytoplasmic tail that is usually involved in activating or inhibiting intracellular signaling through cytoplasmic immunoreceptor tyrosine-based inhibitory or activating motifs [6–8]. Over 20 vertebrate Siglecs have been identified, which are subdivided into two groups based on their common architecture [5]. CD (cluster of differentiation) 22 Siglecs include Siglec-1 (Sialoadhesin), Siglec 2 (CD22), Siglec 4 (myelin-associated glycoprotein, MAG), and Siglec 15 and are structurally conserved among humans, rodents, and most mammals, displaying approximately 25%–30% identity [9]. The second group, CD33 Siglecs, differs according to species and includes Siglec-3, 5, 6, 7, 8, 9, 10, 11, and 14. Within species [10], their extracellular regions have high sequence similarity (50%–85%) and frequently contain conserved tyrosine-based signaling motifs in their cytoplasmic regions [9].

Recent studies have reported spatial and temporal-specific expression in sperm and within the male reproductive tract (MRT) in various species, including humans, sheep, and cattle [11]. Siglecs 1, 2, 5, 6, 10, and 14 are expressed in human spermatozoa and other species. In particular, Siglec-1 is expressed in the neck and mid-piece of human sperm, whereas in bovine sperm, it is expressed in the acrosomal region, the equatorial band, neck, and mid-piece [11–13]. The sperm neck and mid-piece are associated with energy metabolism and mitochondrial function, and Siglecs could play a role in sensing the external Sia environment within the MRT and female reproductive tract (FRT) and regulating the sperm’s response to it.

Nine Siglec proteins have been described in mice, compared with 15 in humans [14]. Murine Siglecs are historically named differently from other species, even if they are orthologs [9]. Five murine Siglecs, Sialoadhesin (Siglec-1), CD22 (Siglec 2), CD33 (Siglec-3), MAG (Siglec 4), and Siglec 15, are highly conserved in humans (listed with their murine and human equivalent names, respectively). Siglecs E, F, and G are probable paralogs of Siglecs 9, 8, and 10, respectively, but Siglec H is unique to mice [5]. To validate their functional consequences, gene knockout models of all murine Siglecs have been generated [15, 16].

Rat and mouse sperm are structurally equivalent and share an anatomical architecture similar to that of other mammals, including humans [17]. Mouse and rat MRTs are anatomically similar to those of other species, including humans [18, 19]. Sperm are produced in paired testes, undergo development, and are stored in the epididymis and urethra [20]. Male accessory sex glands contribute to the seminal component of the ejaculate during ejaculation, performing a function similar to that in other species, including humans [21, 22]. Mice and rats possess a wide range of accessory sex glands, including seminal vesicles, prostate gland, bulbourethral gland (Cowper’s), ampullary gland, coagulation gland, and preputial gland [23, 24]. Rodent reproductive anatomy and Siglec repertoire are conserved across several species, suggesting that rodents are suitable models to explore the function of Siglecs within the reproductive system.

Protein–protein interaction (PPI) networks are a catalog of physical interactions between proteins and are important in predicting potential cell functions. Protein networks are often conserved across species, which supports the inference that their roles are similar. Siglec PPI networks across different species (e.g., human, mouse, and rat) have not been compared. Knowledge of PPI network conservation in different species and comparison with human Siglec PPIs would facilitate understanding the general conservation of Siglec protein function.

In this study, we focused on Siglec-1 and compared Siglec-1 PPI network conservation among humans, mice, and rats to assess the general conservation of Siglec PPIs across species. In addition, the spatial expression patterns of mouse and rat Siglec-1 on sperm and within the MRT were compared with those of other species to determine if similar patterns of expression were evident in these regions. Taken together, this information is an essential initial stage to assess the suitability of rodents as model systems to explore Siglec function in the reproductive system.

## Materials and Methods

### Ethical approval

All experiments were conducted in accordance with the UCD Animal Research Ethics (AREC) approved protocol number (AREC-Brayden-14-28).

### Animals

We obtained adult male rats (strain Wistar) and mice (strain C56BL/6J) from the Charles River Laboratory or the University College Dublin (UCD) Biomedical Facility. The animals were housed in environmentally controlled humidity and temperature conditions under a 12:12 h light/dark cycle with *ad libitum* access to laboratory chow and filtered water. Animals were euthanized by stunning and cervical dislocation. Tissue samples were collected directly from mice (n = 4) and rats (n = 4) and fixed in 10% formaldehyde for histological processing. Immature sperm was manually extracted from the testis tissue and smeared onto Superfrost^®^ slides (VWR, Dublin, Ireland, Cat. No.631-0108). The slides were air-dried for 10 min, wrapped, and stored at −20°C until required.

### Study period and location

The study was conducted from January to November 2023 at the School of Veterinary Medicine, University College Dublin, Ireland.

### Tissue processing

Male reproductive organs were collected from mature rats (n = 4) and mice (n = 4) within 1 h of dispatch. Tissue was dissected to obtain samples of the seminal vesicle (left and right lobes), prostate gland, coagulation gland (left and right lobes), bulbourethral gland (Cowper’s) (left and right lobes), ampullary gland, and preputial gland in addition to blood samples and spleen as positive control tissue for Siglec-1 staining. Tissue samples were fixed in 10% buffered formalin for 36 h and then processed by dehydration through a series of ascending ethanol solutions (30, 60, 90, 100%), cleared in xylene, and impregnated with paraffin wax to form tissue blocks for sectioning. Suitably oriented tissue blocks (4 μm) were sectioned, and tissue integrity was confirmed by microscopic examination after hematoxylin and eosin staining. Serial sections were prepared and mounted on Superfrost^®^ slides (VWR, Cat. No.631-0108) for immunostaining.

### Immunostaining procedure

Human anti-Siglec-1 antibody (1:20 dilution, Santa Cruz Biotechnology Inc. Dallas, Tx, U.S.A, cat. no. Sc-23594) was used for all immunostaining of tissue and sperm and was detected with a biotinylated polyclonal rabbit anti-goat secondary antibody (1:400; DakoCytomation cat. No. E0466, USA) for chromogenic staining and polyclonal rabbit anti-goat (1:30, fluorescein isothiocyanate (FITC)) (DakoCytomation cat. no. F0250, Bath, United Kingdom) for immunofluorescence staining.

For tissue sections and sperm, chromogenic immunostaining was used to visualize the Siglec-1 antibody staining by light microscopy. Tissue sections were deparaffinized by two changes in xylene and dehydrated in descending concentrations of ethanol. Antigen retrieval was performed to enhance epitope access and immunostaining signal. Slides were washed under tap water for 5 min, transferred to plastic jars containing retrieval buffer (sodium citrate 50 mM, pH 6.0), and boiled for 20 min in a microwave (750 watts, microwave highest power). Slides were allowed to cool for 20 min to room temperature (20°C) and washed under running tap water for 5 min. Slides were then stained identically to the Siglec-1 antibody against Siglecs from mice and rats. Negative controls were performed using secondary antibodies only. Antigen-retrieved tissue sections were treated with normal rabbit serum (NRS) instead of primary antibody, followed by normal incubation with secondary antibody and signal development. The absence of staining confirmed the specificity of the anti-Siglec-1 antibody. Fluorescent immunostaining was used to localize and visualize the expression patterns of Siglec-1 on sperm. Sperm were smeared on slides and left to dry at 20°C for 30 min. Slides were incubated for 20 min at 20°C with NRS (Invitrogen, Thermo-Fisher Scientific, Dublin, Ireland, product code 10510) at a 1:20 dilution. NRSs were then removed, and slides were exposed to human anti-Siglec-1 antibody. Slides were washed 3 times with phosphate-buffered saline for 5 min each time; fluorescent secondary antibody was added at a suitable dilution and incubated for 2 h at 20°C, washed, the mounting medium was added, and covered with a coverslip. A fluorescence microscope (Nikon, Eclipse E400, Japan) was used to examine the slides under a FITC filter at 40× magnification. Siglec-1 expression intensity was scored by two separate individuals according to the following scale: No expression, + Low expression, ++ Strong expression, +++ Very strong expression [25].

### Hematoxylin and eosin staining

Paraffin was removed from the tissue sections by two changes in xylene, followed by rehydration in descending concentrations of ethanol. The slides were washed with tap water for 5 min and immersed in Harris’s alum hematoxylin for 6 min. After differentiation and bluing, they were washed again in tap water for 3 min, immersed in dichromate eosin for 2 min, and washed in running tap water for 5 min. Subsequently, sections were dehydrated in ethanol, cleared in xylene, and mounted on immunostaining (synthetic mounting medium). Light microscopy (Nikon, Labophot-2A) was used to examine tissue sections and digital images were recorded for each tissue section at several locations and various magnifications (10×, 20×, and 40×).

### Network analysis of mouse and rat Siglec-1

Network analysis was performed using several different programs. Basic Local Alignment Search Tool (BLAST) on National Center for Biotechnology Information (https://www.ncbi.nlm.nih.gov/) was employed to determine Siglec-1 sequence similarity between humans, mice, and rats. The STRING Database (https://string-db.org) was used to identify Siglec-1 protein-protein interactions (PPI) networks, and University of California, San Francisco (UCSF) Chimera software (https://www.cgl.ucsf.edu/chimera/) was used to detect Siglec-1 interactions with its network proteins through the formation of hydrogen bonds at 10Å distance. To identify the PPI networks in the STRING database, the protein name (Siglec-1) as input followed by a selection of the specific species (humans, mice, or rats) from the drop-down option. Specific 3D structures of proteins were downloaded from the UniProt database (https://www.uniprot.org/) in the Protein Data Bank format. All 3D structures of proteins were imported into UCSF Chimera software and tested with Siglec-1 (human, mice, and rat) and the corresponding network protein for the number of intermolecular hydrogen bonds formed between the two proteins.

## Results

**Figure-1:**
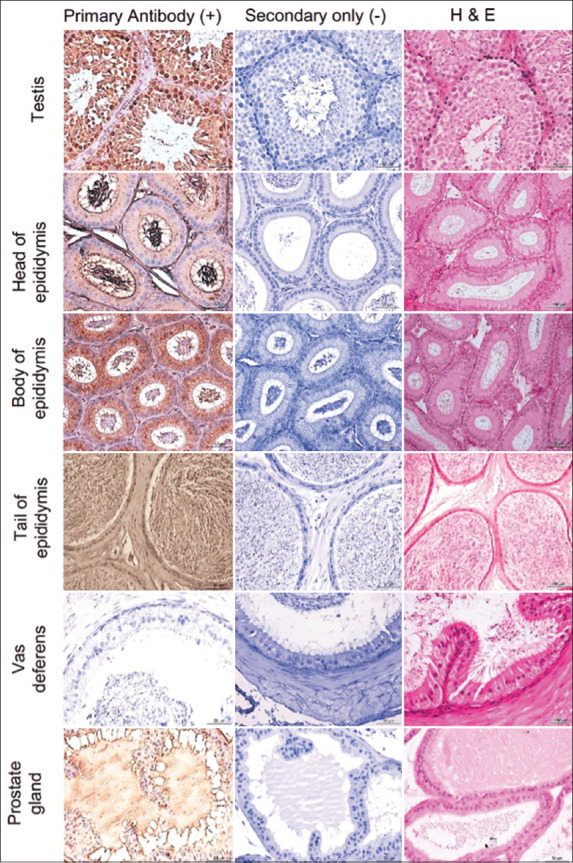
Immunostaining of Siglec-1 in the male reproductive tract and accessory glands of mice using anti-human Siglec-1 antibody. Hematoxylin and eosin staining was used to identify the main structures of MRT and accessory glands of mice. Representative images stained with the primary antibody along with control (stained with secondary antibody) are shown. Siglec-1=Sialic acid-binding immunoglobulin-like lectin 1, MRT=Male reproductive tract.

**Figure-2:**
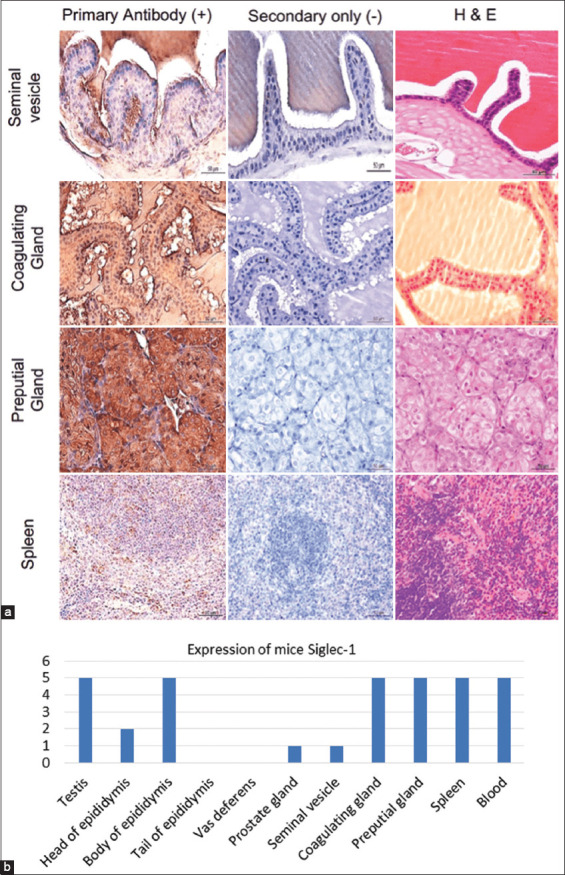
(a) Immunostaining of Siglec-1 in the accessory glands and spleen of mice using human Siglec-1 antibody. Hematoxylin and eosin staining was used to identify the main structures of MRT and accessory glands of mice. Representative images stained with the primary antibody along with control (stained with secondary antibody) are shown. (b) Histogram represents expression levels of mice Siglec-1 in the male reproductive tract, accessory glands, spleen, and blood of mice using human Siglec-1 antibody. Siglec-1=Sialic acid-binding immunoglobulin-like lectin 1, MRT=Male reproductive tract.

**Figure-3:**
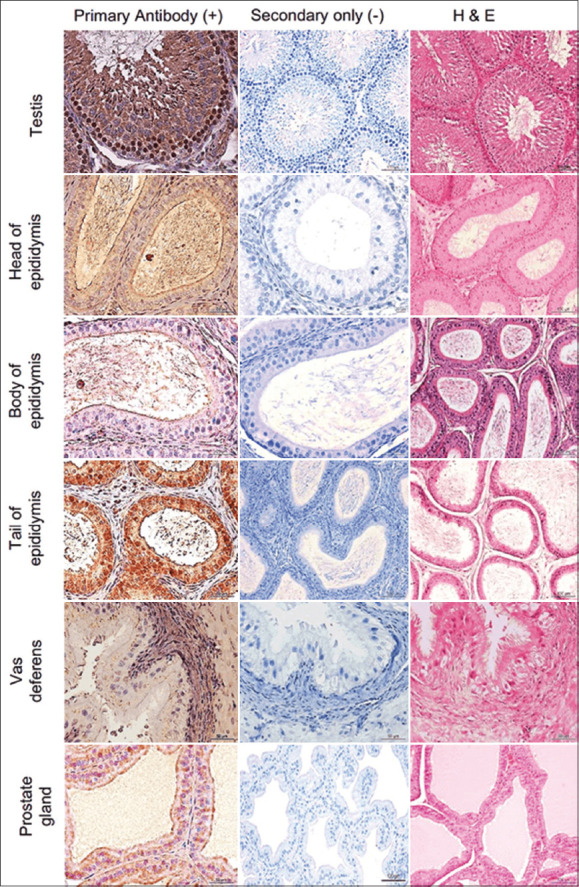
Immunostaining of Siglec-1 in the male reproductive tract and accessory glands of rats using anti-human Siglec-1 antibody. Hematoxylin and eosin staining was used to identify the main structures of MRT of rats and accessory glands. Representative images stained with the primary antibody along with control (stained with secondary antibody) are shown. Siglec-1=Sialic acid-binding immunoglobulin-like lectin 1, MRT=Male reproductive tract.

**Figure-4:**
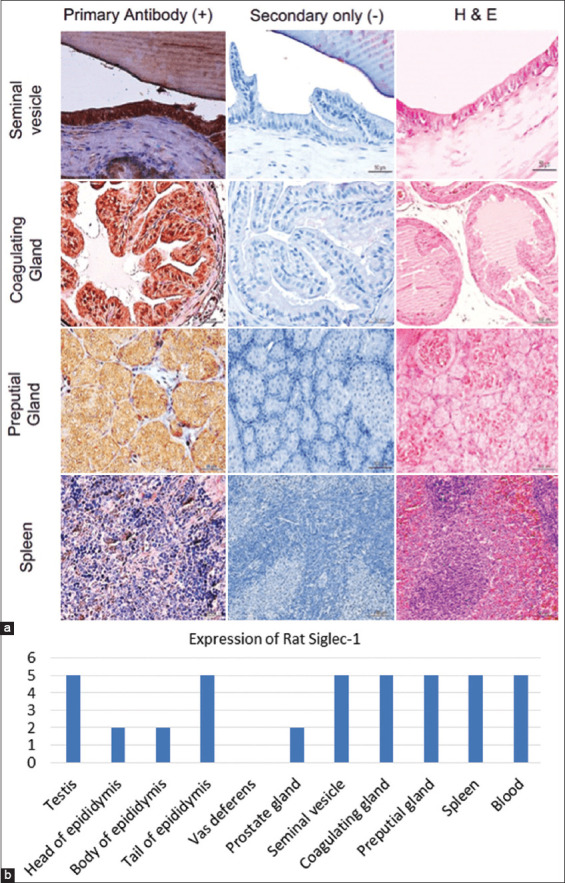
(a) Immunostaining of Siglec-1 in the accessory glands and spleen of rats using human Siglec-1 antibody. Furthermore, hematoxylin and eosin were used to identify the main structures of MRT and accessory glands of rats. Representative images stained with the primary antibody along with control (stained with secondary antibody) are shown. (b) Histogram represents expression levels of rats Siglec-1 in the male reproductive tract, accessory glands, spleen, and blood of mice detected using human Siglec-1 antibody. Siglec-1=Sialic acid-binding immunoglobulin-like lectin 1, MRT=Male reproductive tract.

**Figure-5:**
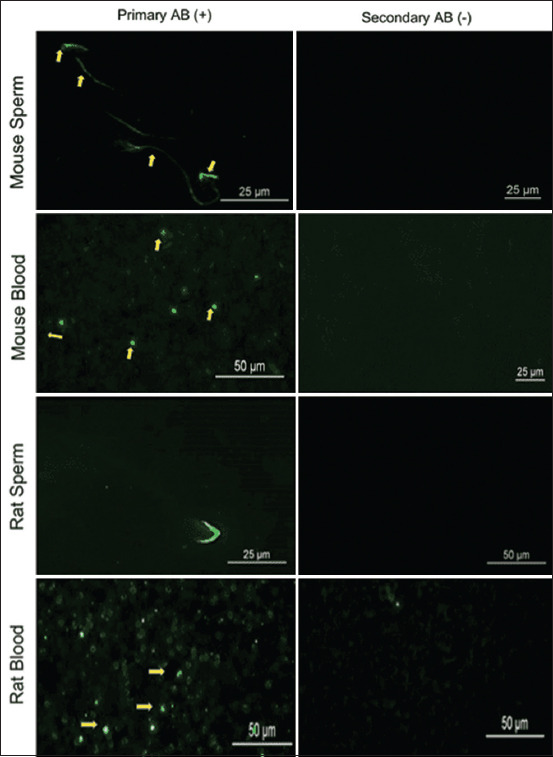
Immunofluorescence-based detection of Siglec-1 (yellow arrows) on sperm and blood smear of mice and rats using human Siglec-1 antibody. The corresponding control (secondary antibody) staining is also shown. Siglec-1=Sialic acid-binding immunoglobulin-like lectin 1.

**Figure-6:**
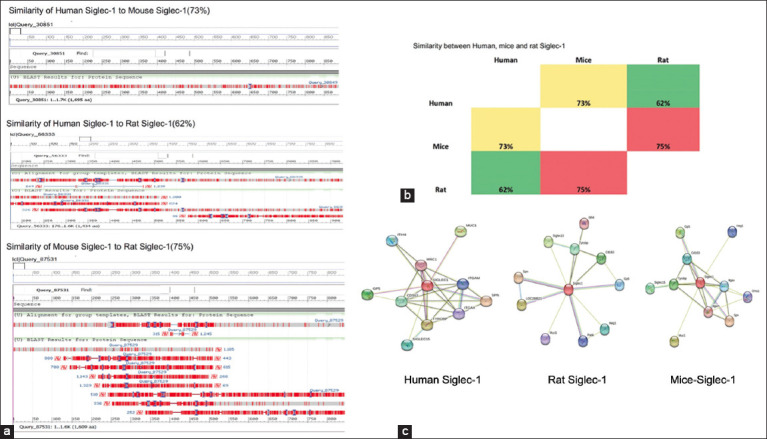
(a) The basic local alignment search tool was used to assess the sequence similarities between the corresponding proteins. (b) The 3D structures of human Siglec-1, mice Siglec-1, and rat Siglec-1 were evaluated for intermolecular interactions using Chimera software by identifying the number of hydrogen bonds formed, and the data are represented as a heatmap. (c) The network proteins of human Siglec-1, mice Siglec-1, and rat Siglec-1 were detected using the STRING database, and the top ten networks were presented. Siglec-1=Sialic acid-binding immunoglobulin-like lectin 1.

Chromogenic and fluorescence staining were used to identify Siglec-1 expression in tissue sections and testicular sperm. Immunofluorescence and chromogenic staining of tissue probed with polyclonal human antibody for Siglec-1 against external epitopes of the protein on tissue and sperm of mice and rats confirmed that human Siglec-1 antibody could cross-react with tissues from both mice and rats (Figures-1–5). Considering the extensive conservation of Siglec-1 between humans, mice, and rats (Figure-6), this cross-reactivity was unsurprising. Rodent leukocytes and spleen tissue were used as positive controls and showed staining of Siglec-1 with human Siglec-1 antibody, which further validates the utility of human Siglec-1 antibody in probing Siglec-1 expression in mice and rats (Figure-5). Positive staining was observed in the presence of primary and secondary antibodies, whereas secondary antibodies alone were not detected. These results confirm that the staining observed was specific to the Siglec-1 antibody.

### Siglec-1 expression in mouse and rat MRT and accessory sex glands

Mouse (n = 4) and rat (n = 4) MRT (seminiferous tubules, rete testis, head, body, and tail of the epididymis and the ductus deferens), as well as accessory sex glands (seminal vesicles, prostate, coagulating, and preputial glands) were probed with human Siglec-1 antibody (Table-1 and Figures-1, 2, and 4), and staining patterns were assessed.

**Table-1:** Summary of Siglec-1 expression patterns in mature mouse and rat male reproductive system and accessory glands (n=4).

Siglec-1 expression	Mouse	Rat
Testis	+++	+++
Head of epididymis	++	++
Body of epididymis	+++	++
Tail of epididymis	−	+++
Vas deferens	−	−
Prostate gland	+	++
Seminal vesicle	+	+++
Coagulating gland	+++	+++
Preputial gland	+++	+++
Spleen	+++	+++
Blood	+++	+++

Siglec-1=Sialic acid-binding immunoglobulin-like lectin 1, + Low expression, ++ Strong Expression, +++ Very strong, and−Negative

Similar Siglec-1 expression patterns were observed in the seminiferous tubule epithelium of mice and rats. Species-specific expression patterns were evident in the epididymis. A similar pattern of Siglec-1 expression was observed in the head and body of the epididymis of both mice and rats, but no Siglec-1 expression was observed in the tail of the epididymis of mice, whereas high Siglec-1 expression was observed in the tail of the epididymis of rats. No Siglec-1 expression was evident in the ductus deferens of either species. Siglec-1 expression within mouse and rat accessory sex gland components also showed species-specific patterns. Siglec-1 expression was observed in the seminal vesicle, prostate, and preputial coagulation glands of both species. Siglec-1 expression in the seminal vesicle of rats was stronger than that in mice.

### Siglec-1 expression in mice and rat sperm

Using fluorescence immunohistochemistry, rat and mouse sperm in testis samples and smears showed similar patterns of Siglec-1 staining (Figure-5 and Table-2). Siglec-1 showed specific staining on the acrosomal region and the mid-piece of sperm of both species, with a higher intensity of staining evident in the rat acrosome and head.

**Table-2:** Summary of Siglec-1 distribution and location on mouse and rat sperm.

Siglec-1 expression	AC	EB	PAC	N	MP	A	T
Sperm of mouse	+	+	-	+	+	+	-
Sperm of rat	+	+	+	-	-	-	-

+=Positive, -=Negative, AC=Acrosomal cap, EB=Equatorial band, PAC=Post-acrosomal cap, N=Neck, MP=Midpiece, A=Annulus, T=Tail

### Analysis of the Siglec-1 network

In this study, we compared the sequences of Siglec-1 in humans, mice, and rats using the protein BLAST and found similarities between species. Mouse and rat Siglec-1 displayed a high degree of similarity (75%), whereas mice and human Siglec-1 showed a similarity of 73.2% and 62.2%, respectively (Figure-6). This result was consistent with our immunostaining results, which demonstrated the expression of human Siglec-1 in mice and rats using a polyclonal antibody for Siglec-1.

PPI network analysis was conducted in two stages. The top 10 proteins associated with Siglec-1 for each species were identified by STRING analysis. The number of hydrogen bonds as a predictor of physical interactions *in vivo* was analyzed for these proteins.

After initial string analysis, six proteins (CD163, integrin alpha-M [ITGAM], platelet glycoprotein V [GP5], mucin-1 (MUC1), Siglec 15, and leukosialin) were present across all species. Hydrogen bond analysis showed that CD163 had the highest potential interaction with Siglec-1 among all species.

At the individual species level, mouse Siglec-1 displayed a strong PPI with several proteins, including the scavenger receptor cysteine-rich type 1 protein M130 (CD163), integrin alpha-X (ITGAX), and ITGAM (Table-3 and Figure-6). In rats, Pas domain-containing serine/threonine kinase (Pask), inter-alpha-trypsin inhibitor heavy chain family member 4 (ITIH4), and CD163 antigen displayed the highest interaction with Siglec-1 (Table-4 and Figure-6), whereas in humans, the top Siglec PPI proteins were macrophage mannose receptor 1, ITIH4, ITGAX, ITGAM, and CD163 (Table-5 and Figure-6).

**Table-3:** Protein–protein interactions of the 3D structure of mouse Siglec-1 with partner proteins.

No.	Proteins	Abbreviation	No. of hydrogen bonds
1	Leukosialin	SPN	3048
2	Mucin 1	Muc1	3987
3	Glycoprotein 5	Gp5	6141
4	TYRO protein tyrosine kinase-binding protein	Tyrobp	1370
5	Sialic acid binding Ig-like lectin 15	Siglec-15	3244
6	Scavenger receptor cysteine-rich type 1 protein M130	CD163	12553
7	Integrin alpha-X	Itgax	10043
8	Protein HEG homolog 1	Heg1	7853
9	Metalloendopeptidase OMA1	Oma1	5935
10	Integrin alpha-M	Itgam	12184

Scale: Red to green denotes high to low number of hydrogen bonds

**Table-4:** Protein–protein interactions of 3D structure of the Siglec-1 in rat with partner proteins.

No.	Proteins	Abbreviation	No. of hydrogen bonds
1	Leukosialin	Spn	3707
2	LOC288521	LOC288521	3247
3	Sialic acid binding Ig-like lectin 15	Siglec-15	4062
4	CD163 antigen	CD163	10153
5	TYRO protein tyrosine kinase-binding protein	Tyrobp	8333
6	Heart development protein with EGF-like domains 1	Heg1	8302
7	Glycoprotein v platelet	Gp5	7703
8	Pas domain containing serine/threonine kinase	Pask	12402
9	Mucin 1	Muc1	7869
10	Inter alpha-trypsin inhibitor	Itih4	11623

Scale: Red to green denotes high to low number of hydrogen bonds

**Table-5:** Protein–protein interactions of 3D structure of the Siglec-1 in human with partner proteins.

No.	Proteins	Abbreviation	No. of hydrogen bonds
1	Leukosialin	SPN	3049
2	TYRO protein tyrosine kinase-binding protein	TYROBP	1221
3	Mucin-1	MUC1	10023
4	Platelet glycoprotein V	GP5	7319
5	Scavenger receptor cysteine-rich type 1 protein M130	CD163	10071
6	Sialic acid-binding Ig-like lectin 15	SIGLEC-15	4740
7	Inter-alpha-trypsin inhibitor heavy chain family member 4	ITIH4	12664
8	Integrin alpha-M	ITGAM	10580
9	Integrin alpha-X	ITGAX	11872
10	Macrophage mannose receptor 1	MRC1	17282

Scale: Red to green denotes high to low number of hydrogen bonds

In general, the PPI Siglec-1 pattern differed across species. CD163 displayed the highest potential level of interaction with Siglec-1 across all species, followed by GP5 at a lower potential level. The network proteins for human and mouse Siglec-1 showed reasonable similarity (Figure-6) with CD 163, ITGAM, and ITGAX, displaying high levels of interaction with Siglec-5 in both species. In contrast, mice and rats had only one protein, CD163, with a high affinity, followed by heart development protein with EGF-like domains 1, with a lower affinity. These data suggest a higher level of PPI conservation between human and mouse Siglec-1 than between mouse and rat or human and rat Siglec-1.

## Discussion

In this study, the expression of Siglec-1 in mouse and rat sperm, MRT tissue, and accessory sex glands of mice and rats was analyzed using an anti-human Siglec-1 antibody. Previous research by Alkhodair *et al*. [11] using an anti-human Siglec-1 antibody detected Siglec-1 expression in human, ovine, and bovine sperm. Similarly, we detected mouse and rat Siglec-1 using anti-human Siglec-1 antibody in this study. A recent study also detected Siglec-5 in the sperm and MRT of camels [26] and in human, ovine, and bovine sperm [11], including Siglec-5, 6, 10, and 14/5. Such cross-species detection of proteins using an antibody generated against human proteins suggests interspecies homology with common antigenic epitopes.

In this study, we identified the expression of Siglec-1 in sperm and within the MRT components of both mice and rats using a human Siglec-1 polyclonal antibody. Our results indicate that mouse Siglec-1 has a pattern similar to that of human Siglec-1 and is expressed in the acrosomal region and head, neck, mid-piece, and annulus regions of the sperm [11]. However, rat Siglec-1 is expressed only in the acrosomal and head regions of the sperm. Therefore, human Siglec-1 is functionally and structurally similar to mouse Siglec-1, whereas rat Siglec-1 is structurally similar to human Siglec-1 and functionally different [9, 27].

In addition, our results confirm that the polyclonal human antibody employed can detect mouse and rat homologs in sperm and MRT tissue, similar to previous reports by Yi *et al*. [28] in other species, including bovine sperm. This cross-reactivity may again be due to extensive interspecies similarity in the Siglec-1 sequence. A similar expression pattern was observed between mice and rat sperm, although some regional variation was observed in expression and relative expression levels. These changes in expression levels may reflect functional differences between these species. Strong Siglec-1 expression was evident on the acrosomal cap of mouse and rat sperm. This finding is consistent with the expression of Siglec-1 on the acrosomal cap in several other species, including humans and cattle. Conservation of this expression on the acrosomal cap suggests a function similar to that observed in other species [11, 28]. The acrosomal cap is involved in capacitation, sperm-zona pellucida binding, and acrosomal exocytosis and stores calcium within the sperm [29, 30]. In humans and bovine species, Sia has been implicated in sperm-oocyte binding and oocyte penetration [31–33], although specific sperm ligands have not been identified. Siglec-1 presents a prominent extracellular structure on the cell surface glycocalyx [5, 34], which may help the acrosomal region of sperm penetrate the secreted fluid within the FRT after mating and facilitate oocyte-sperm fusion. Therefore, it is a potential candidate protein for such interaction. Siglec-1 expression was also observed in the equatorial band and mid-piece of mouse and rat sperm. These regions are involved in sperm motility energy production and calcium storage [35, 36]. It is plausible that Siglec-1 in this region is involved in energy regulation and calcium flux within the sperm in response to external Sia-mediated cues, which influence sperm motility.

It is also possible that Siglecs could interact with Sias on oocytes, suggesting that Siglec-1 is involved in the diversity of sialylated glycans involved in capacitation [8, 37]. Siglec-1 expression in the MRT and accessory sex glands of mice and rats was broadly similar, with some regional and expression level variations evident. In all cases, only very low levels of background staining with secondary antibodies were detected, suggesting that specific staining was responsible for these results. In addition, different levels of staining intensities were observed between tissues and Siglecs, suggesting that different levels of Siglec-1 are present in different areas of the MRT. Siglec-1 has been detected in both species within the seminiferous tubule, and a similar pattern has been observed in cattle [38]. Siglec-1 expression was evident in the head, body, and tail of the rat epididymis, but not in the mouse epididymal tail. Siglec-1 was also expressed in all parts of the human and bovine epididymis, suggesting a species difference between these species and the mouse. Siglec-1 is expressed in the epithelium lining the accessory sex glands, showing some similarities in Siglec-1 expression patterns and differences with bovine expression patterns [28]. Siglec-1 expression in the rat seminal vesicle and prostate glands was higher than that in the mouse.

Previous studies Karmakar *et al*. [39] and Bärenwaldt and Läubli [40] have indicated that Siglecs are usually expressed in immune system cells, including B and T cells, neutrophils, monocytes, and lymphocytes [39, 40]. In addition to the immune system, only limited tissue expression has been described, including placental trophoblasts and brain microglia [41–44].

Expression in male reproductive tissue has not been reported. Judging from the staining patterns we observed, it is unlikely that the results obtained in this study originate from immune-related cells. Expression is largely confined to the seminiferous, epididymal, and glandular epithelium. In addition, the MRT environment is protected from the immune system, which could target differentiated male gametes and immune cells are excluded from the luminal area of the tract [45–47]. Therefore, it is likely that the Siglecs expressed are specifically derived from epithelial and glandular cells within the MRT.

The role of Siglec-1 within MRT components is unknown. The widespread detection of Siglec-1 suggests that it plays a general role in the modulation of Sia function in MRT. The expression pattern of Siglec-1 throughout the components of the MRT also suggests that Siglec-1 shed from specific regions may be absorbed into epithelia and male gametes in other regions. Interestingly, soluble/secreted forms of Siglec-1 have been reported [8, 48, 49]; thus, there is a possibility that soluble versions of Siglec-1, which could be present within the seminal fluid, may play a role within the FRT, perhaps in ligand masking and the mediation of sperm tolerance during passage through FRT.

Siglec-1 PPI analysis in humans, mice, and rats showed that all species share PPI network proteins but suggest a closer structural and functional consensus of mouse and human Siglec-1 PPI networks when compared with rats. The general conservation of Siglec-1 PPI underlines the likely overlap of the function and role of Siglec-1 in different species. The conservation between mouse and human Siglec-1 suggests that the mouse is an appropriate model for translational studies examining the role of Siglec-1 in human reproductive physiology. CD163 displayed the highest potential level of interaction with Siglec-1 across all species. This protein is usually associated with M2 activation [50] and could play a role in sperm tolerance during FRT. Similarly, it acts as an innate immune sensor because it is expressed on macrophages as a macrophage receptor for bacteria [51], which might help sperm in host defense. In addition, ITGAM and ITGAX are highly conserved Siglec-1 network proteins in humans and mice. Immunoglobulin receptors normally regulate the immune response by influencing chemotaxis and migration of immune cells [52, 53]. ITGAM and ITGAX may also play a role in mediating sperm tolerance within FRT. Rat Siglec-1 showed a high degree of interaction with the Pask, which is involved in energy homeostasis and protein translation in cells [54, 55], while its other major network proteins modulate endogenous protease activity in cells [56]. However, a previous study confirmed that it is more related to spermatogenesis upregulation than fertility, sperm production, and motility. These major network proteins suggest that Siglec-1 regulates sperm function during MRT and FRT in rats and that rodents could be a good animal model.

## Conclusion

In summary, Siglec-1 expression was detected in mouse and rat sperm and within discrete MRT components, including the epididymis and several male accessory sex glands of these species. The expression patterns of Siglec-1 in both mice and rats were in broad agreement with those observed in other species, including human and bovine sperm and FRT components. These common sperm and MRT expression patterns suggest the conservation of Siglec-1 structure/function across species. Overall, Siglec-1 network analysis suggests substantial conservation of PPI networks across species, and human and mouse PPI networks are most similar when compared with rat networks. It is likely that Siglec-1 interacts with key network proteins within the MRT and FMT, which may regulate sperm function. Moreover, the conservation of Siglec-1 network proteins between humans and mice suggests that mice could be a good animal model for evaluating Siglec-1 functions in human reproductive physiology. The roles of Siglec-1 in MRT and FRT remain unknown, but it is possible that Siglec-1 mediates sperm Siglec–Sia-dependent interactions and may be involved in specific sperm functions, including immune tolerance, motility, and energy metabolism. A comprehensive analysis of the function of Siglec-1 expression in MRT, FRT, and mouse and rat sperm is required to investigate these potential roles.

## Data Availability

The supplementary data can be available from the corresponding author on a request.

## Authors’ Contributions

HA, AHSK, CR, DK, and JI: Designed the study and reviewed the manuscript. HA, GD, and BH: Conducted the study, implemented the experimental laboratory work, and revised the manuscript. DK, HA, JAI, and CR: Interpreted the data and drafted the manuscript. All authors have read, reviewed, and approved the final manuscript.
